# Assessing Physical Health Status Among Older Adults with Serious Mental Illness

**DOI:** 10.1192/j.eurpsy.2026.10665

**Published:** 2026-07-31

**Authors:** M. Murat Mehmed Ali, D. İpekcioğlu, S. Buzlu, S. Er, M. AvcI, E. Manisali

**Affiliations:** 1 Psychiatric Nursing Department, University of Health Sciences; 2 Institute of Graduate Studies, Istanbul University - Cerrahpaşa; 3 Psychiatry Department, Bakırköy Mazhar Osman Mental Health and Neurological Diseases Education and Research Hospital; 4 Mental Health and Psychiatric Nursing Department, Istanbul University - Cerrahpaşa; 5 Nutrition & Dietetics Department, Biruni University, İstanbul, Türkiye

## Abstract

**Introduction:**

Serious mental disorders, including depression, schizophrenia, and bipolar disorder, have a prevalence of 5.5%, being more common in women. Physical health problems frequently accompany these disorders, increasing mortality and reducing quality of life due to conditions such as cardiovascular disease, diabetes, and obesity. The growing elderly population with mental disorders highlights the importance of addressing their physical health. Yet, inadequate knowledge among healthcare providers complicates access to comprehensive physical care for these individuals. This study aims to examine the physical health status of elderly patients with serious mental disorders.

**Objectives:**

This study aims to comprehensively assess the physical health status of elderly individuals diagnosed with serious mental disorders (such as depression, schizophrenia, and bipolar disorder), by identifying co-occurring medical conditions, evaluating risk factors, and highlighting neglected health parameters that may compromise quality of life and increase mortality risk.

**Methods:**

Seventy-two elderly patients from a psychiatric outpatient clinic in Istanbul participated. Inclusion criteria were DSM-V diagnosis, age 60 or over. Data were collected via face-to-face interviews and physical examinations using the Individual Characteristics Form, Health Improvement Profile, and Mini Nutritional Assessment. Data analysis utilized SPSS v.29.0, with significance at p<0.05.

**Results:**

Participants’ mean age was 68.51±5.67 years; 55.6% were female, and 81.9% unemployed. Mini Nutritional Assessment scores averaged 11.54±1.71, indicating 58.3% had normal nutritional status. Frequently red-flagged parameters included waist circumference (44.1%), blood pressure, blood glucose, dental, eye, breast/prostate examinations, foot care, exercise, and sleep patterns, whereas smoking, alcohol consumption, and substance use were predominantly normal. Green-flagged parameters were body temperature, smoking and alcohol consumption, fluid intake, urinary elimination, bowel habits, substance use, and caffeine intake.

**Conclusions:**

Findings indicate significant physical health risks among elderly psychiatric patients, highlighting neglect in key health parameters. Regular health screening, multidisciplinary follow-up, professional training, individualized counseling, caregiver involvement, and targeted interventions are essential to improve health outcomes.

**Disclosure of Interest:**

None Declared

